# Expressing intentions is not climate action

**DOI:** 10.1073/pnas.2512457122

**Published:** 2025-07-03

**Authors:** Fabian Dablander, Florian Lange, Cameron Brick, Adam R. Aron

**Affiliations:** ^a^Institute for Biodiversity and Ecosystem Dynamics, University of Amsterdam; ^b^Institute for Advanced Study, University of Amsterdam, Amsterdam 1090 GE, The Netherlands; ^c^Behavioral Economics and Engineering Group, Faculty of Economics & Business, Katholieke Universiteit Leuven, Leuven 3000, Belgium; ^d^Department of Psychology, University of Amsterdam, Amsterdam 1001 NK, The Netherlands; ^e^Department of Psychology, University of California San Diego, La Jolla, CA 92093

To secure a livable future, we need substantially more people engaged in climate action. Sinclair et al. (1) present an online study of nearly 8,000 participants with the title “Behavioral interventions motivate action to address climate change,” and with the abstract and significance statement speaking of “motivating people to share news articles and petitions,” “motivating individual actions (e.g., driving less, eating vegetarian meals) and collective actions (e.g., donating, volunteering),” and testing strategies to “motivate people to [...] take action in daily life.”

However, rather than measuring actual behavior, the authors measured intentions to share information about climate change and intentions to engage in proenvironmental behaviors. In the limitations section, they state that “we measured behavioral intentions as opposed to directly observable behavior,” and note that “[b]ehavioral intentions are related to actual behavior.”

We see two major problems. First, a vast literature on the so-called “intention-behavior gap” attests to a poor relationship between intentions and actual behavior (2). Meta-analyses indicate that intentions explain only about 18 to 28% of the variance in actual behavior (3, 4). Empirical studies suggest a large intention-behavior gap for climate action. In one of the very few studies on climate action that measured behavior, a video intervention led to a significant increase in efficacy beliefs, yet only 2% of participants engaged, fleetingly, with real-world action (5). Another study found that only 12.8% of households that intended to implement heat stress reduction methods actually did so (6).

Second, even if the correlation between intentions and actual behavior was much stronger, we cannot expect results from interventions on intentions to generalize to the promotion of actual behavior. To infer effects on behavior from effects on intentions, one would need to establish that these effects are correlated. This has not been established empirically. Given that the costs and benefits of responding to questions about intentions are very different from the costs and benefits of taking climate action in the real world (e.g., participating in protests, becoming vegetarian), such a correlation is unlikely to be sufficiently large (7). This questions the external validity of the results of Sinclair et al.—the relative ordering of the effects in their intention-based intervention tournament is unlikely to be the same when targeting actual behavior.

These two points concern the wider field. A study of almost 16,000 young people found that 52% said they would participate in protests (8), yet our personal experience at protests points to attendance at least a thousand times smaller. And whereas about 11% of people in the United States say they would engage in civil disobedience (9), the actual numbers are also many orders of magnitudes smaller. Similarly, numerous studies rely on intentions to make inferences about the effects of interventions on actual behavior, without providing evidence of a link.

We worry that this paints a deeply misleading picture of climate action, and join calls for the field to move beyond self-reported intentions and measure actual behavior (10).
